# Association of Diet Quality with Depression, Anxiety, and Comorbidity Symptoms in Chinese School-Aged Children

**DOI:** 10.3390/nu17243842

**Published:** 2025-12-09

**Authors:** Yuankai Zhao, Manman Chen, Jiahui Wang, Zichen Ye, Yimin Qu, Zhenghe Wang, Xijie Wang, Yu Jiang

**Affiliations:** 1 School of Population Medicine and Public Health, Chinese Academy of Medical Sciences and Peking Union Medical College, Beijing 100730, China; zhaoyk@pumc.edu.cn (Y.Z.);; 2 School of Health Policy and Management, Chinese Academy of Medical Sciences and Peking Union Medical College, Beijing 100005, China; 3 School of Public Health, Southern Medical University, Guangzhou 510515, China; 4 School of Disaster and Emergency Medicine, Tianjin University, Tianjin 300072, China

**Keywords:** diet quality, mental health, depressive symptoms, anxiety symptoms, school-aged children

## Abstract

**Background:** Depression and anxiety are prevalent mental health disorders among children and adolescents, with diet quality emerging as a modifiable risk factor. However, evidence regarding the association between comprehensive diet quality and mental health in school-aged children remains limited. **Methods:** This cross-sectional study included 400 Chinese children aged 8–12 years. Diet quality was assessed using the low-burden Diet Quality Questionnaire (DQQ), from which three Global Diet Recommendations (GDRs) scores were derived: GDR-Healthy, GDR-Limit, and total GDR. Depression and anxiety symptoms were evaluated using the Children’s Depression Inventory (CDI) and the Social Anxiety Scale for Children (SASC), respectively. Log-binomial regression models were used to estimate risk ratios (RRs) and 95% confidence intervals (CIs) for the associations between GDR scores and mental health symptoms (depression, anxiety, comorbidity). Subgroup analyses stratified by age and sex were conducted to explore heterogeneity. **Results:** Higher total GDR scores were associated with lower risks of depressive symptoms (RR = 0.90, 95% CI: 0.84–0.96), anxiety symptoms (RR = 0.93, 95% CI: 0.88–0.99), and their comorbidity (RR = 0.88, 95% CI: 0.79–0.97) after adjustment for age, sex, zBMI, physical activity, region of residence, only-child status and parental education. The GDR-Healthy score was independently associated with lower risks of depression symptoms (RR = 0.89, 95% CI: 0.83–0.96) and comorbidity (RR = 0.87, 95% CI: 0.79–0.95), while no significant associations between GDR-Limit score and mental health disorders were observed. Subgroup analyses indicated that the association was consistent across sex and age subgroups. **Conclusions:** Better diet quality and particularly higher intake of health-protective foods is associated with lower risks of depression, anxiety, and their comorbidity symptoms in Chinese school-aged children in this cross-sectional study. These findings support the integration of diet quality monitoring and nutritional interventions into public health strategies to promote mental health in children.

## 1. Introduction

Mental health disorders, particularly anxiety and depression, represent a critical global public health challenge among children and adolescents [1]. According to the Global Burden of Disease (GBD) study, an estimated 9.33 million and 38.20 million cases of depression and anxiety, respectively, occurred globally among children aged 5 to 14 years in 2021, with nearly half concentrated in Southeast Asia and Western Pacific regions [2]. School age constitutes a critical developmental period for the onset of mental disorders [1]. Depressive, anxiety, and comorbidity symptoms during this stage adversely affect cognitive development, social functioning, and life quality, while substantially increasing the risk of psychiatric and physical morbidity in adulthood [3,4].

Poor dietary quality leading to unbalanced nutrient intake is a significant modifiable risk factor for mental disorders [5], positioning nutritional medicine as a promising public health strategy for the primary prevention [6]. Associations of specific food consumption with mental health have been widely reported, particularly the protective effects from fruits, vegetables, fish and dietary fiber and the adverse effects from ultra-processed food [7,8,9]. However, research on dietary patterns and composite diet quality scores remains limited and inconsistent. For instance, several studies suggest that anti-inflammatory and Mediterranean dietary patterns are associated with reduced risks of depression and anxiety [10,11,12], and intervention trials based on these patterns demonstrate efficacy [13,14]. Regarding the Healthy Eating Index (HEI) which is aligned with the Dietary Guidelines for Americans, Wang et al. reported that HEI-2015 was associated with a lower depression risk [15], whereas Lassale et al. found no significant association with earlier HEI versions [11].

However, some knowledge gaps still persist. First, existing studies predominantly focus on certain nutrients, specific food groups, or region-specific dietary patterns (e.g., Mediterranean diet), rather than comprehensive, cross-culturally comparable diet quality metrics. Second, most diet quality assessments rely on 24-h recalls or food frequency questionnaires, which are time-consuming and limit widespread applicability. In recent years, the Low-Burden Diet Quality Questionnaire (DQQ) has been developed to rapidly assess the dietary quality and can be adapted across regions and cultures using locally relevant sentinel foods, offering a promising tool for global diet quality monitoring [16,17,18]. Third, although preliminary studies suggest unhealthy dietary patterns elevate mental health risks in youth, research specifically targeting school-aged children remains scarce, which is a critical developmental period for establishing dietary habits and implementing nutritional interventions.

This study aims to investigate associations of the Global Diet Recommendations (GDRs) score based on DQQ with risks of symptoms of depression, anxiety, and their comorbidity among school-aged children. We specifically explore the independent effects of health-protective food intake (GDR-Healthy) and restricted component control (GDR-Limit), while comparing the differences among sex and age groups. The findings will provide evidence to inform diet quality-based strategies for promoting child mental health and help address key evidence gaps in nutritional medicine during this pivotal developmental period.

## 2. Materials and Methods

### 2.1. Study Population

This study baseline data from the Childhood Obesity and New Environmental Factors (CONEF) cohort. The cohort, an ongoing prospective study established by our research team, was initiated in November 2024 in Bengbu, Anhui Province, China, with three-year follow-up assessments planned to comprehensively evaluate dietary patterns, mental health, and anthropometric parameters in school-aged children. Participants were selected through a stratified multi-stage cluster random sampling method. We selected a 12-year consistent school in Bengbu City whose distribution of socioeconomic districts approximates the general composition in China. Five classes from the fourth and fifth grades within this school were randomly selected and all students from these 10 classes were invited to participate. This strategy enhanced the sample’s heterogeneity across social strata, accounting for factors such as family income and parental education.

Eligibility criteria were as follows: (1) Aged 8–12 years. (2) Provided written informed consent from guardians and oral assent from children. (3) Without any severe somatic diseases (cardiac/hepatic/renal disorders, type 1 diabetes), neurodevelopmental conditions (intellectual disability, autism spectrum disorder), or other contraindications.

A total of 462 children were enrolled. After excluding 62 participants who lacked critical data (25 with missing dietary data, 19 with incomplete psychological assessments, and 18 with missing anthropometric measurements), 400 children were included in the final analysis (response rate: 86.6%; Figure S1).

This study was approved by the Institutional Review Board of Tsinghua University (THU-20220013), and written informed consents were obtained from both students and their parents. The study adhered to the principles of the Declaration of Helsinki and complied with the Strengthening the Reporting of Observation Studies in Epidemiology (STROBE) guidelines for observational studies.

### 2.2. Dietary Data Collection and Assessment

Dietary quality data were collected using the low-burden Dietary Quality Questionnaire (DQQ). The DQQ is a rapid assessment tool developed from the World Health Organization (WHO) Healthy Diet framework [19]. It is adapted to sentinel foods (defined as the foods in each food group that were consumed by more than 95% of people) in the Chinese context [17]. The Chinese DQQ comprises 29 food groups to capture dietary intake patterns, with each group assessed through binary (yes/no) response (Table S1). The validity of the DQQ for use in Chinese children and adolescents aged 7–18 has been confirmed, with its sentinel foods shown to capture over 95% of food group consumption in this age group [16]. The Chinese DQQ and technical documentation are accessible via the Global Diet Quality Project website [20].

Three types of GDR score were constructed based on the DQQ: (1) GDR-Healthy score (range 0–9) reflects intake of health-protective foods (fruits, vegetables, whole grains, legumes, dietary fiber) and is aligned with 5 global recommendations; (2) GDR-Limit score (range 0–9) assesses restricted components (total fat, saturated fat, sodium, free sugars, processed meats, unprocessed red meat) as per 6 global guidelines; (3) total GDR score (range −9 to 9) is calculated by subtracting the GDR-Limit score from the GDR-Healthy score, representing overall adherence to 11 dietary recommendations for non-communicable disease prevention. Higher total GDR and GDR-Healthy scores, and lower GDR-Limit scores, reflect better dietary quality.

### 2.3. Mental Health Assessment and Scoring

We used the Children’s Depression Inventory (CDI) to measure depressive symptoms and the Social Anxiety Scale for Children (SASC) to assess anxiety symptoms. If both depression and anxiety symptoms are detected simultaneously, it is defined as the comorbidity of anxiety and depression symptoms.

The CDI consists of 27 items and each item is scored on a three-point Likert scale (0–2). Total scores range from 0 to 54, and scores ≥ 19 defined depressive symptoms in this study [21]. The SASC comprises 10 items and each item uses a dichotomous scoring system (0–1). Total scores range from 0 to 20, with ≥8 indicating potential anxiety symptoms in this study [22]. Both instruments demonstrated good reliability and validity in Chinese pediatric populations [21,22,23].

### 2.4. Covariates

Based on prior evidence [15,24,25], we adjusted for the following covariates: sex (boys/girls), age, BMI z-score (zBMI) (according to the WHO Child Growth Standards in 2016, calculated by the average weight of the normal population of the same age, sex and height [26]), physical activity level (assessed by the International Physical Activity Questionnaire-Short Form (IPAQ-SF), categorized as low/moderate/high groups based on MET-min/week thresholds), region of residence (rural/township/urban), only-child status (yes/no), and education level of fathers and mothers (primary or lower, secondary school, undergraduate).

### 2.5. Statistical Analysis

Descriptive statistics were employed to summarize baseline characteristics of the study participants. Group differences were tested using Mann–Whitney U tests for non-normally distributed continuous variables (presented as median [IQR]), independent *t*-tests for normally distributed continuous variables (presented as mean ± SD), and Pearson’s χ^2^ tests for categorical variables.

In our study, the outcome was not rare, and the estimated odds ratio was not close to the rate ratio (RR). To evaluate the association between GDR scores and mental health symptoms (depression, anxiety, comorbidity), we therefore calculated RRs and 95% confidence intervals (CIs) using log-binomial regression models, or log-Poisson models when log-binomial models did not converge [27]. The RRs and 95% CI were estimated in the three sequential models: Model 1 (unadjusted); Model 2 (adjusted for age, sex, zBMI); Model 3 (further adjusted for physical activity, residence, only-child status, parental education). The GDR scores were analyzed primarily as continuous variables to maximize statistical power and precision. Additionally, to explore potential nonlinear or threshold effects, we also categorized the total GDR score into quartiles for comparative analysis. Restricted cubic splines with four knots (5th, 35th, 65th, 95th percentiles) assessed nonlinear relationships, with significance assessed using Wald χ^2^ tests. Subgroup analyses were stratified by age (≤10 vs. >10 years) and sex (boys vs. girls) [28]. Missing data in the covariates were handled using multiple imputation to preserve the sample size and statistical power.

All analyses were performed using Stata/MP 17.0. Statistical significance was defined as a two-tailed *p* < 0.05.

## 3. Results

### 3.1. Characteristics and Prevalence of Depression and Anxiety Symptoms

Table 1 presents the characteristics of the 400 children included in the final analysis, stratified by comorbidity status of depression and anxiety symptoms. The overall sample comprised 259 boys (64.75%) and 141 girls (35.25%), with a mean age of 10.26 years (SD = 0.63) and a mean zBMI of 0.66 (SD = 1.44). Regarding dietary quality, the mean total GDR score was 0.84 (SD = 2.43), the GDR-Healthy score median was 7.00 (IQR: 5.00, 8.00), and the GDR-Limit score median was 6.00 (IQR: 3.00, 8.00). Among the 400 participants, 113 (28.25%) met criteria for depressive symptoms, 126 (31.50%) for anxiety symptoms, and 71 (17.75%) exhibited comorbid depression and anxiety symptoms.

Compared with those without comorbidity symptoms, the comorbidity symptom group had a significantly higher proportion of girls (47.9% vs. 32.5%, *p* = 0.014), and significantly lower mean of total GDR scores (0.15 ± 2.37 vs. 0.99 ± 2.42, *p* = 0.008) and GDR-Healthy scores (6.00 [IQR: 4.00, 7.00] vs. 7.00 [IQR: 5.00, 8.00], *p* = 0.001). No significant differences were observed between the groups for age, zBMI, physical activity level, region of residence, only-child status, parental education level, or GDR-Limit score (all *p* > 0.05).

**Figure 1:**
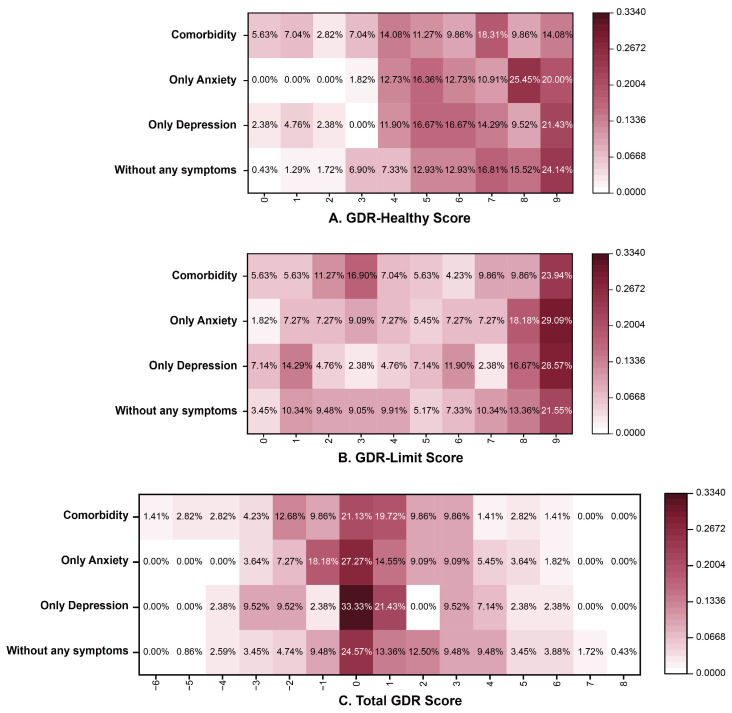
The distribution of GDR scores in different mental health status. Note: GDR (Global Diet Recommendations).

The distribution of GDR scores (stratified by symptom category [without any symptoms, only depression, only anxiety, and comorbidity]) is illustrated in Figure 1. For the GDR-Healthy Score, the highest proportion was at 9 points for the group without any symptoms (24.14%) and the depression only group (21.43%), with the anxiety only group most frequently scoring 8 (25.45%) and the comorbidity group most commonly scoring 7 (18.31%). For the total GDR score, the highest proportion was at 0 points for all groups. For the GDR-Limit score, the highest proportion was at 9 points for all groups.

### 3.2. Association Between GDR Scores and Depression and Anxiety Symptoms

Restricted cubic spline models revealed no significant nonlinear dose–response associations for depression (*P*_non-linear_ = 0.564), anxiety (*P*_non-linear_ = 0.670) or comorbidity symptoms (*P*_non-linear_ = 0.412) (Figure S2).

In the primary analysis treating the score as a continuous variable, each 1-point increment in total GDR score was associated with a 10% decreased risk of depressive symptoms (RR = 0.90, 95% CI: 0.84–0.96; *p* = 0.002) in fully adjusted models. When analyzed by quartiles of GDR score, participants in the highest quartile (Q4) exhibited a 40% lower risk of depression symptoms compared to the lowest quartile (Q1) (RR = 0.60, 95% CI: 0.37–0.98; *p* = 0.040). For anxiety symptoms, each 1-point increase in the continuous GDR score was associated with a 7% lower risk (RR = 0.93, 95% CI: 0.88–0.99; *p* = 0.017), with the Q4 group showing a 40% decreased risk compared to Q1 (RR = 0.60, 95% CI: 0.39–0.93; *p* = 0.021). Regarding comorbidity, every 1-point GDR increase was associated with a 12% reduced risk (RR = 0.88, 95% CI: 0.79–0.97; *p* = 0.012), and the Q4 group demonstrated a 52% lower risk (RR = 0.48, 95% CI: 0.25–0.94; *p* = 0.031) (Table 2).

### 3.3. Association Between GDR-Healthy/GDR-Limit Scores and Depression and Anxiety Symptoms

The total GDR score comprises two distinct subcomponents: GDR-Healthy (reflecting protective food intake) and GDR-Limit (indicating restricted component intake), which were analyzed separately.

In fully adjusted models, each 1-point increase in the continuous GDR-Healthy score was significantly associated with an 11% reduced risk of depressive symptoms (RR = 0.89, 95% CI: 0.83–0.96; *p* = 0.002) and a 13% reduced risk of comorbidity (RR = 0.87, 95% CI: 0.79–0.95; *p* = 0.003), but showed no significant association with anxiety symptoms (*p* = 0.230). Quartile comparisons revealed that participants in the highest GDR-Healthy quartile (Q4) had 42% lower depression symptom risk (RR = 0.58, 95% CI: 0.36–0.93; *p* = 0.025) and 55% reduced comorbidity risk (RR = 0.45, 95% CI: 0.23–0.88; *p* = 0.019) compared to the lowest quartile (Q1).

Conversely, GDR-Limit score showed no significant associations with any outcomes (all *p* > 0.05) (Table 3).

### 3.4. Subgroup Analysis

**Figure 2:**
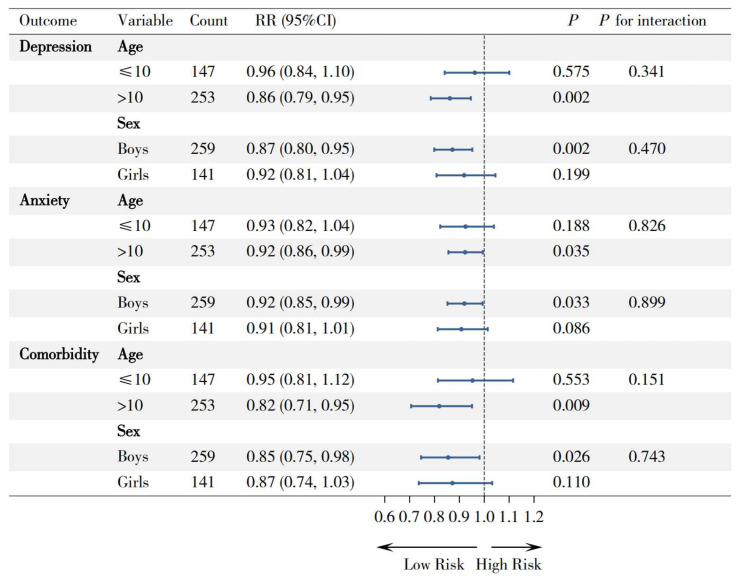
Forest plot for subgroup analyses of the association between GDR score and mental health outcomes, by age and sex. Note: RR (rate ratio), 95% CI (95% confidence interval).

Prespecified subgroup analyses were conducted to examine potential effect modifications by age and sex (Figure 2). The associations between higher GDR scores and reduced risks of depression (RR = 0.86, 95% CI: 0.79–0.95; *p* = 0.002), anxiety (RR = 0.92, 95% CI: 0.86–0.99; *p* = 0.035), and comorbidity symptoms (RR = 0.82, 95% CI: 0.71–0.95; *p* = 0.009) were significant in children older than 10 years, whereas no significant associations were observed in those aged ≤ 10 years (all *p* > 0.05). Similarly, while protective associations were significant among boys for all symptoms (depression: RR = 0.87, *p* = 0.002; anxiety: RR = 0.92, *p* = 0.033; comorbidity: RR = 0.86, *p* = 0.026), the associations among girls were not statistically significant (all *p* > 0.05).

However, all interaction tests were nonsignificant (all *p* for interaction > 0.05), indicating consistent protective directionality of GDR effects across subgroups despite variations in magnitude.

## 4. Discussion

This study demonstrates significant associations between diet quality assessed by GDR scores (derived from the DQQ) and depression, anxiety, and their comorbidity symptoms among Chinese school-aged children. Specifically, a higher total GDR score, reflecting better overall diet quality, was consistently associated with lower risks of depressive, anxiety, and their comorbidity symptoms in both sexes. Notably, these protective associations were primarily linked to GDR-Healthy component (reflecting protective foods), rather than GDR-Limit components.

Depression and anxiety symptoms are significant mental health issues, and their comorbidity symptoms are relatively common, which affected nearly 20% of our participants. Previous studies have reported that compared to a single symptom, depression–anxiety comorbidity significantly increased the risk of suicidal ideation and attempts and had a poorer response to treatment [29], indicating that it deserves particular concern in early intervention. Our current findings extended previous studies by adding evidence for inverse associations of the diet quality assessed by total GDR score with depression, anxiety and especially comorbidities in school-aged children. In this pivotal stage, dietary behavior not only has a significant impact on physical and mental development, but is also highly malleable and could develop into lifelong healthy behaviors [30]. Public health strategies that integrate dietary quality monitoring and promotion for children in both family and school settings represent a low-cost, promising approach worthy of further investigation, based on the observed associations.

Notably, when further examining the effects of the components of total GDR, the GDR-Healthy score, which captures intake of health-protective foods such as fruits, vegetables, whole grains, legumes, and dietary fiber, were significantly associated with reduced risks of depression and comorbidity. This aligns with existing evidence highlighting the protective roles of these food groups against mental disorders. Potential mechanisms may involve the beneficial changes in the richness and diversity of the intestinal microbiota (e.g., dietary fiber promoting the growth of bifidobacterium, Lactobacillus, etc., and leading to a reduction in Enterobacteriaceae) [31,32], anti-inflammatory and antioxidant effects (omega-3 polyunsaturated fats in fish and polyphenols rich in fruits and vegetables) [33], and neurofunctional regulation (for example, B vitamins from whole grains can modulate neurotransmitters and neuropeptides, thereby influencing the brain activities related to mood and appetite) [34]. In contrast, the GDR-Limit scores, which reflect intake of items such as total fats, saturated fats, sodium, free sugars, processed meats, and unprocessed red meat, showed no significant associations with any mental health outcomes in this population. This may suggest that observed associations between better mental health and diet quality in children are primarily explained by the inclusion of protective foods rather than the restriction of unhealthy components, although longer-term exposure may be needed to observe effects of dietary restrictions. The nutritional education and public health strategies should focus on providing access to various nutritious, health-protective foods for school-aged children.

The associations between diet, depression and anxiety have garnered considerable attention in recent years. Evidence has established associations between certain dietary metrics (such as the Healthy Eating Index and Mediterranean diet scores) and mental health [24,35]. However, existing metrics remain culturally specific to Western populations, lacking global cross-cultural applicability and feasibility [36]. In our study, we used the GDR scores from the China-adapted DQQ to assess diet quality. Building on the widely practical Minimum Dietary Diversity for Women (MDD-W), which uses food groups to proxy for quantitative dietary intake, the DQQ incorporates additional food group-based indicators focused on non-communicable disease (NCD) risk factors and reflects the WHO Healthy Diet Fact Sheet (2018) [19,37]. Crucially, it can be adjusted through sentinel foods to take the diversity of different cultural contexts, locally available foods, and dietary customs into account [19,37,38]. Furthermore, the DQQ is rapid, easy to understand, and low-cost (reducing the cost to ~USD 1 per respondent, which is 500 times less than a quantitative survey [39]). These features make the DQQ particularly suitable for large-scale surveys of children and general population, as well as for comparing diet quality at the national and global levels.

In addition, our subgroup analyses revealed the protective associations were consistent across sex and age subgroups, despite being more pronounced in boys and older age subgroups. This sex difference may be attributed to distinct metabolic demands and dietary behaviors in adolescent males, who typically exhibit higher caloric intake but lower diet diversity than females, potentially exacerbating neurocognitive vulnerabilities during pubertal development [40]. Children approaching adolescence (≥10 years) demonstrate increased dietary autonomy while facing academic stress escalation, in which nutritional deficiencies may disproportionately impact neurological development and functional regulation [30,41]. However, since the formal interaction tests were not significant, these findings must be considered preliminary and hypothesis-generating. These findings need to be further validated in larger populations to clarify subgroup-specific effects.

There are several limitations in our study. Firstly, the cross-sectional design limits determination of the causal relationship of dietary quality with mental health. We cannot determine whether poor diet quality precedes the onset of depression/anxiety symptoms or whether pre-existing mental health conditions lead to less healthy dietary choices due to reduced motivation or appetite. Although the diets of school-aged children are primarily determined by familial and school provisions, which potentially reduces the above concern, prospective studies remain essential to account for unmeasured confounders and establish temporal sequence. Second, residual confounding from unmeasured factors is possible. Although we adjusted for most major confounders identified in the prior literature, key variables such as household income and parental cooking practices were not measured and adjusted for, which could bias the estimates. Third, self-reported data on mental health symptoms are subject to potential reporting and social desirability biases, meaning children might underreport symptoms due to the desire to act in an acceptable and positive manner. Fourth, the DQQ captures dietary patterns through binary self-reporting and does not quantify specific nutrient or energy intake, thus it cannot replace quantitative methods like 24 h recalls. However, its low cost and rapid administration make it a highly feasible tool for large-scale epidemiological studies where assessing overall diet quality is the primary objective [19]. Finally, the generalizability of our findings to the broader Chinese population may be limited because of the single-region sample. Future multi-center studies are needed to validate these findings across diverse populations in China.

## 5. Conclusions

This study provides the first population-based study on the associations between dietary quality assessed by the DQQ and two major mental health disorders in school-aged children. The findings show that children with higher dietary quality were associated with lower risks of both depression and anxiety symptoms and their comorbidity in this cross-sectional study. Notably, these protective associations were primarily linked to the intake of health-protective foods (including fruits, vegetables, whole grains, legumes and dietary fiber). The findings support further investigation into the potential of dietary interventions to complement existing mental health prevention efforts, especially in individuals with comorbidity symptoms. Public health policies could consider school- and community-based nutrition programs to improve dietary diversity and mental well-being in children, particularly in resource-limited settings.

## Figures and Tables

**Table 1:** Characteristics of participants by comorbidity status of anxiety and depression symptoms.

		Total (*N* = 400)	Without Comorbidity Symptoms (*N* = 329)	Comorbid Anxiety–Depression (*N* = 71)	*p*-Value
Sex (%)	Boys	259 (64.75)	222 (67.48)	37 (52.11)	0.014 *
	Girls	141 (35.25)	107 (32.52)	34 (47.89)	
Age (SD)		10.26 ± 0.63	10.28 ± 0.64	10.21 ± 0.60	0.425
zBMI (SD)		0.66 ± 1.44	0.69 ± 1.42	0.54 ± 1.52	0.422
Physical activity level (%)	Low	142 (35.50)	110 (33.43)	32 (45.07)	0.109
	Moderate	85 (21.25)	75 (22.80)	10 (14.08)	
	High	173 (43.25)	144 (43.77)	29 (40.85)	
Region of residence (%)	Rural	68 (17.00)	51 (15.50)	17 (23.94)	0.189
	Township	75 (18.75)	61 (18.54)	14 (19.72)	
	Urban	257 (64.25)	217 (65.96)	40 (56.34)	
Only child (%)	Yes	132 (33.00)	102 (31.00)	30 (42.25)	0.067
	No	268 (67.00)	227 (69.00)	41 (57.75)	
Education level of mother (%)	Primary school or below	41 (10.25)	34 (10.33)	7 (9.86)	0.976
	Secondary school	228 (57.00)	188 (57.14)	40 (56.34)	
	Undergraduate	131 (32.75)	107 (32.52)	24 (33.80)	
Education level of father (%)	Primary school or below	55 (13.75)	41 (12.46)	14 (19.72)	0.223
	Secondary school	223 (55.75)	184 (55.93)	39 (54.93)	
	Undergraduate	122 (30.50)	104 (31.61)	18 (25.35)	
GDR score (SD)		0.84 ± 2.43	0.99 ± 2.42	0.15 ± 2.37	0.008
GDR-Healthy score (IQR)		7.00 (5.00, 8.00)	7.00 (5.00, 8.00)	6.00 (4.00, 7.00)	0.001 *
GDR-Limit score (IQR)		6.00 (3.00, 8.00)	6.00 (3.00, 8.00)	5.00 (3.00, 8.00)	0.526

Note: Continuous data are presented as mean ± SD (normally distributed) or median (IQR) (nor-normally distributed). Categorical variables are presented as frequency (%). * *p* < 0.05; Comparisons: Independent *t*-tests for normally distributed continuous variables; Mann–Whitney U tests for non-normally distributed continuous variables; Pearson’s χ^2^ tests for categorical variables. Abbreviations: zBMI (Body Mass Index z-score), GDR (Global Diet Recommendations), SD (Standard Deviation), IQR (Inter Quartile Range).

**Table 2:** Associations between GDR scores and depression and anxiety symptoms.

		Model 1		Model 2		Model 3	
		RR (95% CI)	*p*-Value	RR (95% CI)	*p*-Value	RR (95% CI)	*p*-Value
Depressive symptoms							
	Quartile 1	1.00 (Ref)		1.00 (Ref)		1.00 (Ref)	
	Quartile 2	0.84 (0.55, 1.26)	0.393	0.84 (0.56, 1.26)	0.403	0.86 (0.57, 1.29)	0.466
	Quartile 3	0.85 (0.56, 1.27)	0.427	0.87 (0.58, 1.33)	0.527	0.86 (0.57, 1.30)	0.474
	Quartile 4	0.60 (0.37, 0.97)	0.036 *	0.60 (0.37, 0.98)	0.040 *	0.60 (0.37, 0.98)	0.040 *
	Continuous	0.90 (0.85, 0.96)	0.002 *	0.90 (0.84, 0.96)	0.002 *	0.90 (0.84, 0.96)	0.002 *
Anxiety symptoms							
	Quartile 1	1.00 (Ref)		1.00 (Ref)		1.00 (Ref)	
	Quartile 2	0.74 (0.50, 1.08)	0.117	0.73 (0.49, 1.08)	0.118	0.77 (0.52, 1.15)	0.208
	Quartile 3	0.82 (0.57, 1.18)	0.278	0.84 (0.58, 1.20)	0.338	0.85 (0.59, 1.22)	0.386
	Quartile 4	0.56 (0.36, 0.87)	0.010 *	0.57 (0.37, 0.88)	0.011 *	0.60 (0.39, 0.93)	0.021 *
	Continuous	0.93 (0.88, 0.98)	0.007 *	0.92 (0.87, 0.98)	0.007 *	0.93 (0.88, 0.99)	0.017 *
Comorbidity							
	Quartile 1	1.00 (Ref)		1.00 (Ref)		1.00 (Ref)	
	Quartile 2	0.61 (0.34, 1,10)	0.100	0.62 (0.35, 1.12)	0.115	0.64 (0.35, 1.16)	0.140
	Quartile 3	0.84 (0.50, 1.41)	0.512	0.89 (0.53, 1.49)	0.646	0.85 (0.51, 1.43)	0.539
	Quartile 4	0.47 (0.24, 0.90)	0.023 *	0.48 (0.25, 0.92)	0.026 *	0.48 (0.25, 0.94)	0.031 *
	Continuous	0.88 (0.81, 0.97)	0.008 *	0.88 (0.80, 0.97)	0.010 *	0.88 (0.79, 0.97)	0.012 *

Mode 1 included GDR score as the sole variable; Model 2 further controlled for age, sex and zBMI; Model 3 additionally controlled for physical activity, region of residence, only-child status, parental education. * *p* < 0.05.

**Table 3:** Associations of GDR sub-scores with depression and anxiety symptoms.

		Model 1		Model 2		Model 3	
		RR (95% CI)	*p*-Value	RR (95% CI)	*p*-Value	RR (95% CI)	*p*-Value
GDR-Healthy score							
Depression symptoms							
	Quartile 1	1.00 (Ref)		1.00 (Ref)		1.00 (Ref)	
	Quartile 2	0.66 (0.44, 0.99)	0.044 *	0.66 (0.44, 0.99)	0.042 *	0.69 (0.46, 1.05)	0.086
	Quartile 3	0.58 (0.39, 0.86)	0.007 *	0.59 (0.39, 0.88)	0.010 *	0.63 (0.41, 0.95)	0.029 *
	Quartile 4	0.53 (0.33, 0.85)	0.008 *	0.54 (0.34, 0.86)	0.010 *	0.58 (0.36, 0.93)	0.025 *
	Continuous	0.87 (0.82, 0.94)	<0.001 *	0.88 (0.82, 0.94)	<0.001 *	0.89 (0.83, 0.96)	0.002 *
Anxiety symptoms							
	Quartile 1	1.00 (Ref)		1.00 (Ref)		1.00 (Ref)	
	Quartile 2	0.73 (0.49, 1.08)	0.116	0.71 (0.48, 1.05)	0.085	0.79 (0.52, 1.19)	0.254
	Quartile 3	0.79 (0.55, 1.14)	0.206	0.81 (0.57, 1.16)	0.256	0.87 (0.60, 1.25)	0.454
	Quartile 4	0.60 (0.38, 0.95)	0.029 *	0.61 (0.39, 0.97)	0.038 *	0.69 (0.43, 1.13)	0.139
	Continuous	0.93 (0.88, 0.99)	0.034 *	0.94 (0.89, 1.00)	0.065	0.96 (0.90, 1.03)	0.230
Comorbidity							
	Quartile 1	1.00 (Ref)		1.00 (Ref)		1.00 (Ref)	
	Quartile 2	0.46 (0.26, 0.81)	0.008 *	0.45 (0.26, 0.79)	0.006 *	0.51 (0.28, 0.94)	0.030 *
	Quartile 3	0.52 (0.31, 0.86)	0.012 *	0.54 (0.32, 0.92)	0.022 *	0.58 (0.35, 0.98)	0.041 *
	Quartile 4	0.38 (0.19, 0.73)	0.004 *	0.40 (0.20, 0.77)	0.006 *	0.45 (0.23, 0.88)	0.019 *
	Continuous	0.84 (0.77, 0.92)	<0.001 *	0.85 (0.78, 0.93)	0.001 *	0.87 (0.79, 0.95)	0.003 *
GDR-Limit score							
Depression symptoms							
	Quartile 1	1.00 (Ref)		1.00 (Ref)		1.00 (Ref)	
	Quartile 2	0.95 (0.60, 1.48)	0.814	0.92 (0.58, 1.45)	0.720	0.91 (0.58, 1.44)	0.702
	Quartile 3	0.83 (0.54, 1.30)	0.420	0.83 (0.54, 1.30)	0.424	0.87 (0.56, 1.35)	0.543
	Quartile 4	1.02 (0.66, 1.58)	0.938	1.02 (0.66, 1.58)	0.918	1.07 (0.70, 1.66)	0.748
	Continuous	0.99 (0.94, 1.04)	0.695	0.99 (0.94, 1.05)	0.761	1.00 (0.95, 1.06)	0.987
Anxiety symptoms							
	Quartile 1	1.00 (Ref)		1.00 (Ref)		1.00 (Ref)	
	Quartile 2	1.25 (0.81, 1.93)	0.311	1.24 (0.81, 1.91)	0.324	1.29 (0.83, 2.00)	0.251
	Quartile 3	1.05 (0.68, 1.62)	0.826	1.06 (0.69, 1.64)	0.779	1.09 (0.71, 1.68)	0.695
	Quartile 4	1.25 (0.81, 1.93)	0.311	1.27 (0.83, 1.96)	0.270	1.33 (0.88, 2.03)	0.176
	Continuous	1.01 (0.97, 1.06)	0.596	1.02 (0.97, 1.07)	0.480	1.02 (0.98, 1.07)	0.355
Comorbidity							
	Quartile 1	1.00 (Ref)		1.00 (Ref)		1.00 (Ref)	
	Quartile 2	1.24 (0.69, 2.23)	0.464	1.20 (0.66, 2.16)	0.552	1.21 (0.66, 2.22)	0.547
	Quartile 3	0.80 (0.43, 1.49)	0.477	0.81 (0.44, 1.51)	0.516	0.86 (0.46, 1.59)	0.629
	Quartile 4	1.01 (0.54, 1.87)	0.983	1.04 (0.56, 1.92)	0.906	1.09 (0.59, 1.99)	0.789
	Continuous	0.97 (0.91, 1.04)	0.447	0.98 (0.92, 1.05)	0.578	0.99 (0.92, 1.06)	0.735

Note: Mode 1 includes GDR Sub-score as the sole variable; Model 2 further controlled for age, sex and zBMI; Model 3 additionally controlled for physical activity, region of residence, only-child status, parental education. * *p* < 0.05.

## Data Availability

The original contributions presented in this study are included in the article/Supplementary Materials. Further inquiries can be directed to the corresponding authors.

## Supplementary Materials

The following supporting information can be downloaded at https://www.mdpi.com/article/10.3390/nu17243842/s1, Table S1: Diet scores constructed from the China Diet Quality Questionnaire (DQQ); Figure S1: Sampling flow chart of the Bengbu School Children Obesity Cohort project; Figure S2: Restricted cubic spline analyses of the association between GDR score and mental health outcomes.
