# Supplementary material for: Association of Diet Quality with Depression, Anxiety, and Comorbidity Symptoms in Chinese School-Aged Children
Source: Nutrients. 2025 Dec 9;17(24):3842. doi: 10.3390/nu17243842 (PMC12735964; doi:10.3390/nu17243842)
Supplement: Supplementary file 1 [file nutrients-17-03842-s001.zip › nutrients-4007114-supplementary.pdf]

Supplementary material

Table S1. Diet scores constructed from the China Diet Quality Questionnaire (DQQ).

Food Group	Questions	MDD-W/ Dietary Diversity Score	GDR-Healthy (0 to 9)	GDR-Limit (0 to 9)	Total GDR (-9 to 9)
	Yesterday, did you eat any of the following foods:				
**1. Staple foods made from grains**	Rice, noodles, steamed buns, or bread?<br>米饭；面条；馒头；面包				
**2. Whole grain**	Corn, cornmeal, oats, millet, barley, brown rice, or black rice?<br>玉米（鲜）；玉米面；燕麦片；小米；大麦/糌粑；糙米；黑米；全麦面包	√	√		√
**3. White root/tubers**	Potato, lotus root, starch noodles, yam, taro, or turnip?<br>马铃薯；藕（莲藕）；粉丝/粉条；山药；芋头；大头菜				
**4. Legumes**	Bean curd or tofu, bean curd sheet, soybean milk, soybeans, or other dried beans? 豆腐；豆腐皮；豆浆；黄豆；其他干豆类	√	√		√
	Yesterday, did you eat any of the following vegetables:				
**5. Vitamin A-rich orange vegetables**	Carrots, pumpkin or butternut squash, or sweet potatoes that are orange inside? 胡萝卜；南瓜；红薯	**5/8** √	√		√
**6.1 Dark green leafy vegetables**	Chinese cabbage, water spinach, Chinese spinach, rape, bok choy, sweet potato leaves, or broccoli?<br>大白菜；空心菜；菠菜；油菜；小白菜；番薯叶；西兰花	√	√		√
**6.2 Dark green leafy vegetables**	Mustard leaves, chrysanthemum leaves, radish leaves, amaranth leaves, beet leaves, or watercress?<br>芥菜；茼蒿；萝卜叶；苋菜；甜菜叶；西洋菜				
**7.1 Other vegetables**	Cabbage, tomatoes, eggplant, loofah, green beans, local celery, or cucumber?<br>包菜/圆白菜；番茄；茄子；丝瓜；四季豆；芹菜；黄瓜	√	√		√
**7.2 Other vegetables**	Mushrooms, lettuce, radish, cauliflower, seaweed, bamboo shoot, bell pepper, or bean sprout? 蘑菇；生菜；萝卜；菜花；紫菜；笋；甜椒/柿子椒；豆芽				
	Yesterday, did you eat any of the following fruits:				
**8. Vitamin A-rich fruits**	Persimmon, cantaloupe, ripe mango, passion fruit, fresh or dried apricots, or papaya? 柿子；哈密瓜；芒果；百香果；杏或杏干；木瓜	**5/8** √	√		√
**9. Citrus**	Orange, tangerine, pomelo , grapefruit, or kumquat?<br>橙；橘子/柑橘；柚子；西柚；金桔		√		√
**10.1 Other fruits**	Apple, pear, watermelon, banana, grapes, kiwi, or dragonfruit?<br>苹果；梨；西瓜；香蕉；葡萄；猕猴桃；火龙果	√	√		√
**10.2 Other fruits**	Jujube, longan, wampee, lychee, pomegranate, cherries, or peaches?<br>枣子；龙眼；黄皮果；荔枝；石榴；樱桃；桃				
	Yesterday, did you eat any of the following sweets:				
**11. Grain-baked sweets**	Cakes, cookies, sweet pastries, mooncake, rice dumplings, or egg tart?<br>蛋糕；甜饼干；甜糕点；月饼；甜粽子/汤圆；蛋挞			√	√
**12. Other sweets**	Candy, chocolates, jelly pudding, ice cream, or popsicles?<br>糖果；巧克力；果冻；冰激凌；棒冰			√	√
	Yesterday, did you eat any of the following foods of animal origin:				
**13. Eggs**	Chicken eggs, preserved duck eggs, quail eggs, pigeon eggs, or goose eggs?<br>鸡蛋；咸鸭蛋（白）/松花蛋（黑）；鹌鹑蛋；鸽子蛋；鹅蛋	√			
**14. Cheese**	Cheese?<br>奶酪	**14/15/25** √			
**15. Yogurt**	Yogurt?<br>酸奶	**14/15/25** √			
**16. Processed meat**	Sausages, bacon, ham, larou, luncheon meat, beef jerky, processed beef product, or pork jerk? 腊肠；培根；火腿；腊肉；午餐肉；牛肉干；酱牛肉；猪肉脯			√	√
**17. Unprocessed red meat (ruminant)**	Beef, lamb sheep or goat, or organs from cow or sheep?<br>牛肉；羊肉；驴肉；马肉；内脏 (牛、羊、驴、马)			√	√
**18. Unprocessed red meat (non-ruminant)**	Pork, or pig organs?<br>猪肉；猪内脏	√			
**19. Poultry**	Chicken, duck, goose, pigeon, or gizzard?<br>鸡；鸭；鹅；鸽子；鸡胗				
**20. Fish and seafood**	Fish or seafood?<br>鱼；海鲜				

	Yesterday, did you eat any of the following other foods:				
**21. Nuts and seeds**	Sunflower seeds, pumpkin seeds, watermelon seeds, peanuts, chestnuts, walnuts, almonds, or sesame paste?<br>葵花子；南瓜子；西瓜子；花生；栗子；核桃；杏仁；芝麻酱	√	√		√
**22. Packaged ultra-processed salty snakes**	Chips such as Lays, Pringles, Doritos, shrimp chips, macaroni crisp, or spicy strip? 薯片（如乐事、品客等）；多力多滋；虾条；通心脆；辣条			√	√
**23. Instant noodles**	Instant noodles or instant rice noodles?<br>方便面；速食米粉			√	√
**24. Deep fried foods**	French fries, fried bread stick, fried pancake, fried dough twist, fried glutinous rice ball, fried bean curd, chicken nugget, or deep fried meat?<br>薯条；油条；油饼；麻花；炸糕；炸豆腐；鸡块；炸肉			√	√
	Yesterday, did you have any of the following beverages:				
**25. Fluid milk**	Milk or milk powder?<br>牛奶；奶粉	**14/15/25** √			
**26. Sweet tea/coffee/milk drinks**	Flavored milk, milk tea / bubble tea, Nutri-Express, Yakult, bottled tea beverage, or coffee with sugar?<br>果味奶；奶茶/珍珠奶茶；营养快线；酸乳饮料；瓶装茶饮料；加糖咖啡				
**27. Fruit juice**	Fruit juice or fruit juice beverage?<br>果汁；果汁饮料				
**28. Sugar-sweetened beverages**	Soft drinks such as Coca-Cola, Pepsi, Fanta, Sprite, energy drinks, or sports drinks? 软饮料，如可口可乐，百事可乐，芬达，雪碧等；运动饮料；能量饮料			√	√
	Yesterday, did you get food from any place like...				
**29. Fast food**	KFC, McDonald's, Pizza Hut, Burger King, Subway, or Dicos?<br>肯德基；麦当劳；必胜客；汉堡王；赛百味；德克士			√	√

Note: The Chinese DQQ and technical documentation are accessible via the Global Diet Quality Project website https://www.dietquality.org/countries/chn.

If participants ate or drank any one in the food group of each question, it would be recorded as "yes".

MDD-W: Minimum Dietary Diversity for Women; GDR: Global Dietary Recommendations scores.

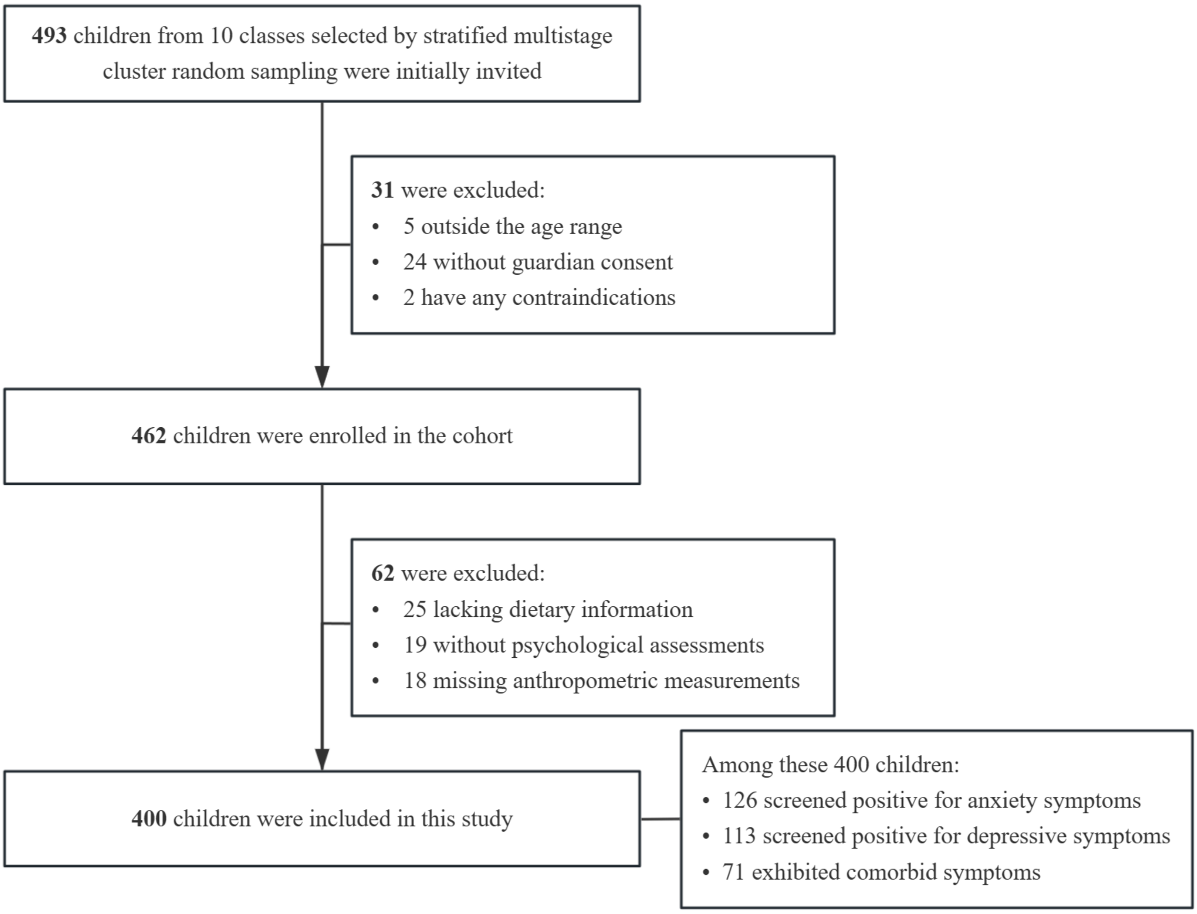

Figure S1 Sampling flow chart of the Bengbu School Children Obesity Cohort project

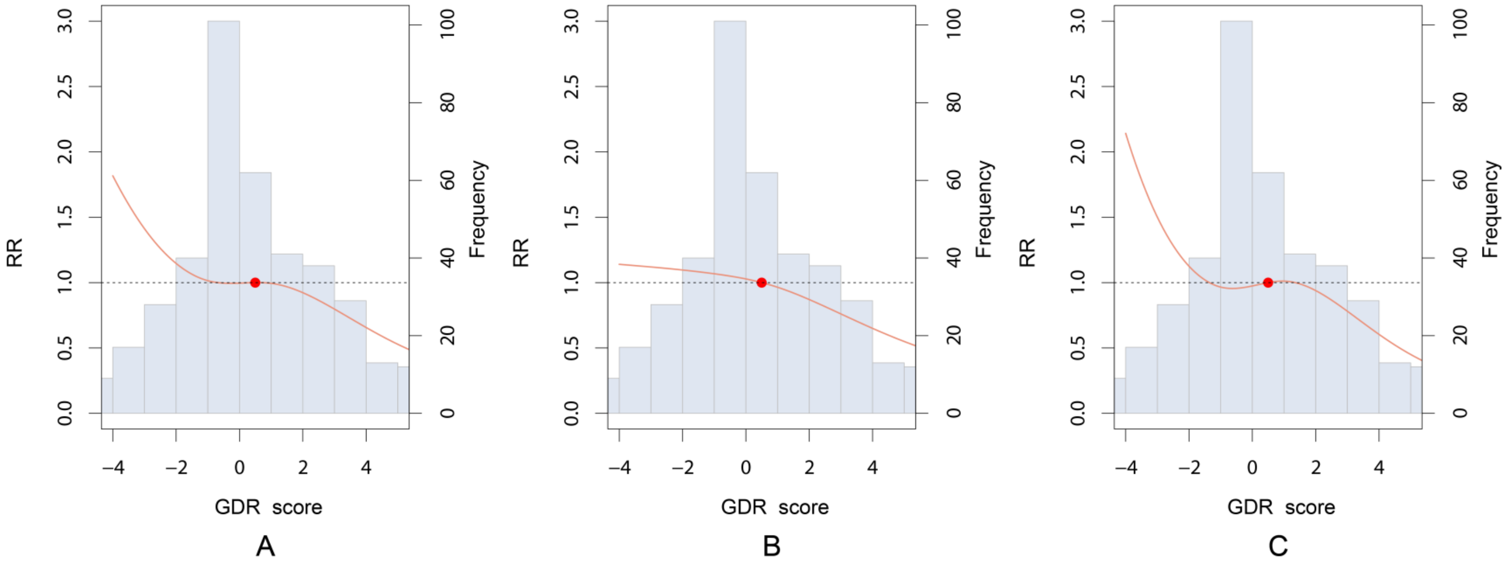

Figure S2. Restricted cubic spline analyses of the association between GDR score and mental health outcomes.

Note: A. Depression symptoms, B. Anxiety symptoms, C. Comorbidity

RR (rate ratio).
